# A food-exchange model for achieving the recommended dietary intakes for saturated fat in Irish children: analysis from the cross-sectional National Children’s Food Survey II

**DOI:** 10.1017/S1368980024000971

**Published:** 2024-05-03

**Authors:** Aileen O’Connor, Maria Buffini, Anne P Nugent, Laura Kehoe, Albert Flynn, Janette Walton, John Kearney, Breige McNulty

**Affiliations:** 1 Institute of Food and Health, School of Agriculture & Food Science, University College Dublin, Belfield, Dublin 4, Ireland; 2 Institute for Global Food Security, Queen’s University Belfast, Northern Ireland; 3 Department of Biological Sciences, Munster Technological University, Cork, Ireland; 4 Department of Biological Sciences, Cork Institute of Technology, Cork, Ireland; 5 School of Biological and Health Sciences, Technological University Dublin, Dublin, Ireland

**Keywords:** SFA, Dairy, Food-exchange, Children

## Abstract

**Objective:**

To identify the main foods determining SFA intakes and model the impact of food exchanges to improve compliance with dietary fat recommendations in Irish children.

**Design:**

Estimated food and nutrient intakes were obtained from a cross-sectional study, the National Children’s Food Survey II. Participants were categorised into low, medium and high SFA consumers, and the contribution of food categories to SFA intakes was compared. A food-exchange model was developed, whereby a selected range of high SFA foods was exchanged with lower SFA or unsaturated fat alternatives.

**Setting:**

Participants were randomly selected from primary schools throughout the Republic of Ireland.

**Participants:**

A representative sample of 600 Irish children (5–12 years).

**Results:**

The main determinants of low and high SFA consumers were milk, cheese and butter. These foods, including snack foods and meat and meat products, were considered exchangeable foods within the model. Compared with baseline data, modelled intakes for total fat, SFA, MUFA and *trans*-fat presented decreases of 3·2, 2·7, 1·6 and < 0·1 % of total energy (% TE), respectively. PUFA, *n*-6, *n*-3 and alpha-linolenic acid showed increases of 1·0, 0·8, 0·2 and 0·1 % TE, respectively. Compliance with total fat, MUFA and *trans*-fat recommendations remained adequate (100 %). Adherence to SFA and PUFA recommendations improved from 18 to 63 % and 80 to 100 %, respectively.

**Conclusion:**

The food-exchange model decreased SFA intakes and increased PUFA intakes, suggesting modest dietary changes to children’s diets can effectively improve their overall dietary fat profile.

CVD encompasses a number of interrelated diseases involving the heart and blood vessels such as CHD, myocardial infarction, stroke and peripheral vascular disease^(1)^. It represents 31 % of all global deaths, making it the leading cause of death in the world^(2,3)^. Although CVD predominantly manifests in adulthood, the asymptomatic phase of its development can begin as early as childhood^(4)^. Early studies by McGill Jr *et al.* ^(5)^ and Berenson *et al.* ^(6)^ examined the presence of fatty streak lesions in children and adolescents, detecting advanced levels of atherosclerosis, another common risk factor for CVD. They discovered these lesions increased significantly in those presenting elevated levels of BMI, systolic blood pressure, serum TAG and LDL cholesterol concentrations, thus, indicating that children can be predisposed to CVD risk without presenting any obvious manifestations in early years.

Dietary fat is associated with several chronic disease risks, including CVD^(7)^. Furthermore, well-established evidence indicates that elevated intakes of SFA can have negative implications for cardiovascular health^(7)^. However, research suggests that the replacement of SFA with unsaturated fats could reduce the risk and occurrence of CVD^(8,9)^. While evidence on SFA substitution with MUFA remains insufficient, adequate evidence of SFA replacement with PUFA has been reported^(8,9)^. In response to this, dietary fat recommendations have been developed advising populations to limit SFA intakes while increasing unsaturated fat intakes^(10–12)^. This has been recognised as an essential strategy to reduce the incidence of CVD, among other diet-related chronic diseases^(10–12)^. Despite this approach, SFA and PUFA intakes in children remain inadequate globally^(13–15)^. In Ireland, current SFA and PUFA intake recommendations are ≤ 10 % TE and ≥ 6 % TE, respectively^(9,16)^. Yet, adherence to both recommendations in Irish school-aged children has shown only slight improvements since 2005^(17)^. In 2005, the percentage of Irish children complying with SFA recommendations was extremely low at 4 % but improved slightly to 7 % in 2019. Within the same period, adherence to PUFA recommendations improved from 35 % to 71 %^(17)^. Although PUFA improvements were significantly better than those reported for SFA, progress is still required^(17)^.

When contemplating a targeted approach to population nutrition, the importance of considering all factors known to influence diet quality has been highlighted, and a combination of strategies to combat various dietary issues is needed^(18,19)^. Along with dietary recommendations, a number of product reformulations and food replacement strategies have been proposed to improve the population’s dietary fat intake profiles^(20–24)^. Reformulation efforts have previously been applied to dairy products through the alteration of bovine feeding practices, whereby dairy cows are fed high MUFA and PUFA diets, thus, modifying the fatty acid composition of the milk produced^(20,25)^. Significant reductions in LDL and total cholesterol levels following the human consumption of such modified milk and dairy products have been reported, suggesting dietary fat modification measures at an agricultural level could be useful for improving cardiovascular health^(26–29)^. Nevertheless, the cost of animal feed constitutes a large proportion of the total cost in the agricultural sector, and dietary modifications at this level could be costly and difficult to implement^(30)^. In addition, comparisons between feeding systems tend to encourage milk produced from grass-fed over non-grass-fed cows due to cost efficiency and more favourable fatty acid profiles such as higher levels of conjugated linoleic acid^(31)^. One must also consider the quality of reformulation practices in terms of sustainability. Dietary sustainability plays an essential role in reducing global environmental pressures and impacts^(32)^. Therefore, any reformulation efforts to improve diet quality should be developed through sustainable food systems. Likewise, when recommending food replacements as a strategy to improve diet quality, sustainable dietary patterns need to be considered to ensure minimal environmental impact while maintaining nutritional adequacy^(33)^.

Intervention studies such as the Dietary Intervention and Vascular Function (DIVAS) study and the LIPGENE project (‘Diet, genomics and the metabolic syndrome: an integrated nutrition, agro-food, social and economic analysis’) have investigated alternative food replacement strategies such as food-exchange models in adult cohorts, successfully modifying dietary fat intakes without altering other components of the diet, thus, providing minimal disruption to participants dietary habits^(10,34)^. Shaw *et al.* define this as ‘exchangeable’ fat, whereby the fat that is not intrinsic within a food product can be easily removed and replaced from the diet^(34)^. In addition, these flexible modelling approaches enable participants to make practical and sustainable dietary substitutions, improving their dietary fat profile with minimal effort. However, if these strategies were implemented at a population level, they would rely heavily on personal choice and responsibility^(19)^. Nevertheless, a number of approaches are required to achieve overall improvements in dietary intake^(18)^. Hence, food-exchange models could be a valuable tool to complement existing dietary guidelines, further contributing to improvements in diet quality.

Strategy tools such as food-exchange modelling to improve dietary fat intake in child populations and evidence of their efficacy are scant. It is essential that children and their parents/guardians are provided with practical dietary advice relevant to their everyday dietary habits^(35)^. Hence, when developing food-exchange strategies to decrease SFA intakes, it is important to identify the dietary factors that influence fat type with respect to the population group. Therefore, the purpose of this present study is to first identify the key sources and dietary determinants of SFA intakes in Irish children and, second, to examine dietary modification approaches influenced by food-exchange models such as identifying the dietary sources of exchangeable fat, in order to improve SFA intakes.

## Methods

### Study design and populations

The present analysis was based on the cross-sectional National Children’s Food Survey II which examined the habitual food and beverage intake of children residing in the Republic of Ireland. A total of 600 children (300 boys, 300 girls) aged 5–12 years took part in the survey which was conducted between April 2017 and May 2018. The selection process was based on the 2016 national population census data^(36)^, and participants were recruited from primary schools drawn from the Department of Education and Skills’ database^(37)^, with the only exclusion criteria being a sibling to another participant. The overall response rate was 65 %, and the analysis of the demographic profile of participants demonstrated a representative sample of Irish children with respect to age, sex and geographical location^(36)^. A statistical weighting was applied for social class to account for non-response bias where appropriate.

### Dietary data collection and analysis

A consecutive 4-d, semi-weighed food diary was used to collect food and beverage intake data. Typically, the parents and/or guardians were asked to record detailed information regarding the amount and types of all foods, beverages and nutritional supplements consumed by the child over the 4-d recording period. However, some older children recorded their own consumption with the assistance of their parents and/or guardians. To account for differences in children’s eating habits between week and weekend days, the 4-d food diary required the inclusion of at least one weekend day. A food quantification protocol was also established by the Irish Universities Nutrition Alliance and adapted for National Children’s Food Survey II^(38)^. Additional information such as cooking methods, brand names of foods, recipe details, eating locations and participant definitions of eating occasions were recorded. Food intake data were entered into the nutritional software package Nutritics V5.02 (Nutritics, Dublin, Ireland). This data was coupled with additional food composition data from McCance and Widdowson’s *The Composition of Foods*, sixth^(39)^ and seventh^(40)^ editions to calculate nutrient intake. A food consumption database was generated from the exported data, listing each individual food item consumed by participants. Each food item was recorded with its corresponding nutrient composition. All food and beverages consumed were aggregated into one of twenty-six food categories according to their predominant fat profile or associated food-based dietary guidelines, based on previous groupings published for the Irish adult population with modifications made to account for the exclusion of alcohol^(41)^.

### Dietary determinants of SFA intakes

The dietary determinants of SFA intakes in Irish children were investigated using tertile analysis. Tertiles were generated based on the percentage of total energy (% TE) from SFA intakes, and participants were split into their respective low, medium and high consumer categories. The contribution of food categories to intakes (% TE) across the tertiles was established, and the differences between the consumer groups were identified.

### Food-exchange model and dietary modifications

A food-exchange model was established to evaluate potential improvements to the dietary fat profile in Irish children. It was based on previously developed, flexible food-exchange models from a number of intervention studies that aimed to identify dietary sources of exchangeable fat that would not impact total energy or other macronutrient intakes^(10,42–44)^. The model used in this study was largely based on the major contributors of SFA intakes in Irish children and current food-based dietary guidelines for Ireland^(45)^. It was important that the sources were easily accessible within the diet or exchangeable. Based on the main SFA contributors and food-based dietary guidelines, Table 1 displays the selected food sources of exchangeable fat, which included specific dairy products, meat products and some snack foods. The model exchanged selected high SFA exchangeable foods with lower SFA and/or higher unsaturated fat alternatives commonly consumed in this sample of Irish children or common in the Irish market. Whole milk and cheese were replaced with low-fat alternatives; fresh and processed meat products were replaced with leaner cuts, grilled or lower fat alternatives. Butter and butter spreads were replaced with a high PUFA style spread. In addition, all biscuits including cookies were replaced with a lower SFA ‘plain’ alternative, whereas popcorn, cream crackers and rice cakes were substituted with lower fat alternatives. No changes were made to the actual amounts of food consumed, only the food types. Following the selected dietary modifications, the modelled mean daily intakes were generated and compared with baseline intakes.

**Table 1:** Exchangeable foods used in SFA replacement model

Baseline foods	Replacement foods
Whole milk	Low-fat/semi-skimmed milk
Full-fat cheese	Reduced fat cheese
Butter/butter spreads	High PUFA spreads
Snacks*	Low-fat alternatives
Fresh meat	Lean cuts, grilled not fried
Meat products	Low-fat alternatives, meat with fat trimmed, grilled not fried

* Includes biscuits, cookies, cream crackers, rice cakes and popcorn only.

### Statistical analyses

Statistical analyses were conducted using SPSS® V24.0 for Windows™ (SPSS Inc.). For tertile analysis, one-way ANOVA with Scheffe *post hoc* test was utilised to assess differences between the groups. Descriptive statistics including mean and sd were calculated for daily total fat, SFA, MUFA, PUFA, *n*-6, *n*-3, *trans*-fat, alpha-linolenic acid (ALA), EPA and DHA intakes (g/d and % TE) for the total population. The percentage contribution from each food category to total fat and fatty acid constituents was also calculated. Paired sample *t* tests were utilised to compare fatty acid intakes between baseline and modelled intakes. All were adjusted using the Bonferroni correction method for multiple comparisons. Compliance with the UK^(9,45)^ and the European Food Safety Authority’s^(13)^ dietary fat intake recommendations were assessed using the approach described by Wearne and Day^(46)^. Statistical significance for all analyses in this study was classified as *P* ≤ 0·05.

## Results

### Percentage contribution of food categories to SFA intakes

The percentage of the population was split into tertiles of the lowest, medium and highest SFA consumers. Their mean daily intakes of SFA (% TE) are as follows: 11·1 % (lowest), 14·0 % (medium) and 17·0 % (highest). Mean daily intakes of all other macro- and micronutrients are reported in online supplementary material, Supplemental Table 1. The percentage contribution of major food categories to mean daily SFA intakes (% TE) across tertiles is presented in Table 2. The percentage contribution of ‘whole milk’ (15 %), ‘cheeses’ (10 %) and ‘butter’ (7 %) was significantly higher in the highest SFA consumers compared with the lowest SFA consumers (8, 5 and 4 %, respectively). In the highest SFA consumers, ‘fresh meat’; ‘low-fat, skimmed and fortified milks’; ‘potatoes chipped, fried and roasted’; and ‘bread and rolls’ provided significantly lower contributions to total energy compared with the lowest SFA consumers (*P* ≤ 0·05).

**Table 2:** Comparison of the percentage contribution of food categories between tertiles of SFA intakes (% TE) in Irish children

	SFA						
	Lowest consumers (*n* 200)		Medium consumers (*n* 200)		Highest consumers (*n* 200)		*P* *
	Mean	sd	Mean	sd	Mean	sd	
SFA (% TE)	11·1^a^	1·37	14·0^b^	0·66	17·0^c^	1·68	<0·001
Food categories (%)							
Fresh meat	5·7^a^		4·6^ab^		3·6^b^		<0·001
Meat dishes	5·7		6·2		5·6		1·000
Meat products	7·2		6·8		7·3		1·000
Whole milk	8·4^a^		12·6^b^		14·7^b^		<0·001
Low-fat, skimmed and fortified milks	3·3^a^		2·2^ab^		1·2^b^		<0·001
Cheeses	5·2^a^		8·0^b^		10·3^c^		<0·001
Butter	3·5^a^		5·6^b^		7·0^b^		<0·001
Low-fat spreads	0·3		0·2		0·1		1·000
Spreading fats and oils	4·2^a^		3·2^ab^		2·8^b^		0·728
Ice cream and creams	3·1		2·6		3·7		1·000
Puddings and chilled desserts	4·8		4·6		4·8		1·000
Savouries	5·9		4·5		3·6		0·338
Savoury snacks	2·6		2·0		1·8		1·000
Soups, sauces and miscellaneous foods	1·6		1·7		1·5		1·000
Biscuits, cakes pastries and buns	12·3		11·8		12·2		1·000
Sugars, confectionery and preserves	8·3^a^		7·2^ab^		6·2^b^		0·858
Potatoes†	1·3^a^		1·2^ab^		0·7^b^		0·182
Potatoes chipped, fried and roasted	2·4^a^		1·4^b^		1·0^b^		<0·001
Potato products	0·5^a^		0·2^ab^		0·2^b^		0·546
Fish, fish products and fish dishes	1·5^a^		0·9^ab^		0·8^b^		1·000
Eggs and egg dishes	1·5		1·8		2·3		1·000
Vegetable and pulse dishes	0·3		0·5		0·4		1·000
Breads and rolls	4·4^a^		3·9^a^		2·8^b^		<0·001
RTEBC	1·5^a^		1·2^ab^		0·9^b^		0·520
Other‡	4·5		5·0		4·5		1·000
Nutritional supplements	0·0		0·0		0·0		1·000

*n*, number; % TE, percentage of total energy; RTEBC, ready-to-eat breakfast cereals.

* One-way ANOVA was used with Scheffe *post hoc* test to assess the difference between tertiles; adjusted *P* value using Bonferroni correction method for multiple comparisons.

† Potatoes food category includes boiled, baked and mashed.

‡ Other food categories include rice, pasta, flours, grains, other breakfast cereals, non-alcoholic beverages, nuts and seeds, herbs and spices, fruit, vegetables, other milk and milk-based beverages.

^abc^Different superscript letters indicate significant differences in mean values across tertiles (*P* < 0·05).

### Comparison of baseline and modelled intakes of energy, dietary fat and other nutrients

Comparisons and the percentage difference between baseline and modelled intakes of energy, total fat and constituent fatty acids (g/d and % TE) are displayed in Table 3. As expected, significant changes were observed across the majority of fatty acids. Total fat, SFA, MUFA and *trans*-fat presented significant decreases of 3·2, 2·7, 1·6 and 0·08 % TE, respectively, whereas for PUFA, *n*-6, *n*-3 and ALA, significant increases of 0·97, 0·78, 0·24 and 0·05 % TE, respectively, were noted. There was a small but significant increase in DHA (0·001 % TE) but no significant change in EPA. Energy intakes (kcal/d) also decreased significantly in the modelled intakes (*P* < 0·001). Likewise, similar patterns to the total population (% TE) were apparent when examined by g/d and when split by sex (data not shown). When assessing the overall nutrient profiles between both baseline and modelled intakes, no differences were observed across macronutrient intakes when measured in g/d. When measured as % TE, protein and total sugar were significantly higher in the modelled diet (16·8, 19·3 %, respectively) compared with baseline (15·9, 18·8 %, respectively; *P* < 0·001). No differences were noted across micronutrient intakes, with the exception of vitamin E, which significantly increased from 6·87 µg/d at baseline to 8·10 µg/d (Table 4).

**Table 3:** Comparison of energy, total fat (g/d and % TE) and its constituent fatty acids in Irish children between baseline and food-exchange modelled intakes

	Total population					
	Baseline		Model			
	(*n* 600)		(*n* 600)			
	Mean	sd	Mean	sd	*P* *	% Δ
Energy (kcal/d)	1502	364	1442	348	<0·001	−4·0
Total fat (g/d)	56·4	17·9	49·1	15·9	<0·001	−13·0
SFA (g/d)	23·5	7·76	18·2	6·12	<0·001	−22·3
MUFA (g/d)	23·4	7·98	20·0	7·04	<0·001	−14·5
PUFA (g/d)	9·41	3·55	10·6	4·16	<0·001	12·5
*n*-6 (g/d)	6·11	2·33	7·11	2·91	<0·001	16·5
*n*-3 (g/d)	1·29	0·91	1·62	1·05	<0·001	26·0
*Trans*-fat (g/d)	0·81	0·38	0·66	0·30	<0·001	−18·4
ALA (g/d)	1·05	0·59	1·09	0·61	<0·001	4·4
EPA (mg/d)	63·1	259	60·5	260	<0·001	−4·2
DHA (mg/d)	80·4	284	78·6	284	<0·001	−2·3
Total fat (% TE)	33·6	5·04	30·4	4·94	<0·001	−9·4
SFA (% TE)	14·0	2·76	11·3	2·27	<0·001	−19·1
MUFA (% TE)	13·9	2·48	12·3	2·40	<0·001	−11·0
PUFA (% TE)	5·59	1·31	6·56	1·75	<0·001	17·4
*n*-6 (% TE)	3·63	0·89	4·41	1·30	<0·001	21·6
*n*-3 (% TE)	0·76	0·38	1·00	0·49	<0·001	31·8
*Trans*-fat (% TE)	0·49	0·19	0·41	0·16	<0·001	−15·0
ALA (% TE)	0·62	0·29	0·67	0·30	<0·001	8·6
EPA (% TE)	0·036	0·10	0·036	0·11	0·184	−0·6
DHA (% TE)	0·046	0·11	0·047	0·12	<0·001	1·3

*n*, number of participants; Δ, change/difference; g/d, grams per day; % TE, percentage of total energy; ALA, alpha-linolenic acid.

* Paired samples *t* test for comparison of means between baseline and the model.

**Table 4:** Comparison of the macro- and micronutrient intakes in Irish children between baseline and food-exchange model for the total population

	Total population				
	Baseline		Model		
	(*n* 600)		(*n* 600)		
	Mean	sd	Mean	sd	*P* *
Macronutrients					
Protein (g/d)	59·5	17·5	60·2	17·7	1·000
Carbohydrate (g/d)	200	49·0	200	49·2	1·000
Total sugar (g/d)	75·1	25·9	74·0	25·7	1·000
Fibre (g/d)	13·9	4·53	13·9	4·55	1·000
Protein (% TE)	15·9	2·78	16·8	3·01	<0·001
Carbohydrate (% TE)	50·0	5·38	52·3	5·29	1·000
Total sugar (% TE)	18·8	4·96	19·3	5·08	<0·001
Micronutrients					
Vitamin A (µg)	644	435	627	429	1·000
Vitamin C (mg)	76·0	108	76·0	108	1·000
Vitamin D (µg)	4·44	5·82	4·74	5·84	1·000
Vitamin E (µg)	6·87	3·80	8·10	4·03	<0·001
Vitamin B_6_ (mg)	1·54	0·79	1·54	0·79	1·000
Vitamin B_12_ (µg)	4·64	2·32	4·64	2·33	1·000
Thiamine (mg)	1·41	0·63	1·42	0·63	1·000
Riboflavin (mg)	1·59	0·67	1·61	0·68	1·000
Total folate (µg)	206	80·3	209	80·9	1·000
DFE (µg)	248	120	251	121	1·000
Total niacin (mg)	29·1	9·34	29·2	9·36	1·000
Na (mg)	1701	523	1705	522	1·000
K (mg)	2003	585	2005	584	1·000
Ca (mg)	790	290	800	293	1·000
Mg (mg)	191	57·2	192	57·3	1·000
P (mg)	1002	304	1008	307	1·000
Fe (mg)	9·01	3·11	9·04	3·11	1·000
Zn (mg)	7·23	2·48	7·16	2·45	1·000

*n*, number of participants; DFE, dietary folate equivalents.

* Paired sample *t* test for comparison of means between baseline and model (*P* < 0·05); adjusted *P* value using Bonferroni correction method for multiple comparisons.

### Percentage contribution of exchangeable food categories to dietary fat

**Fig. 1:**
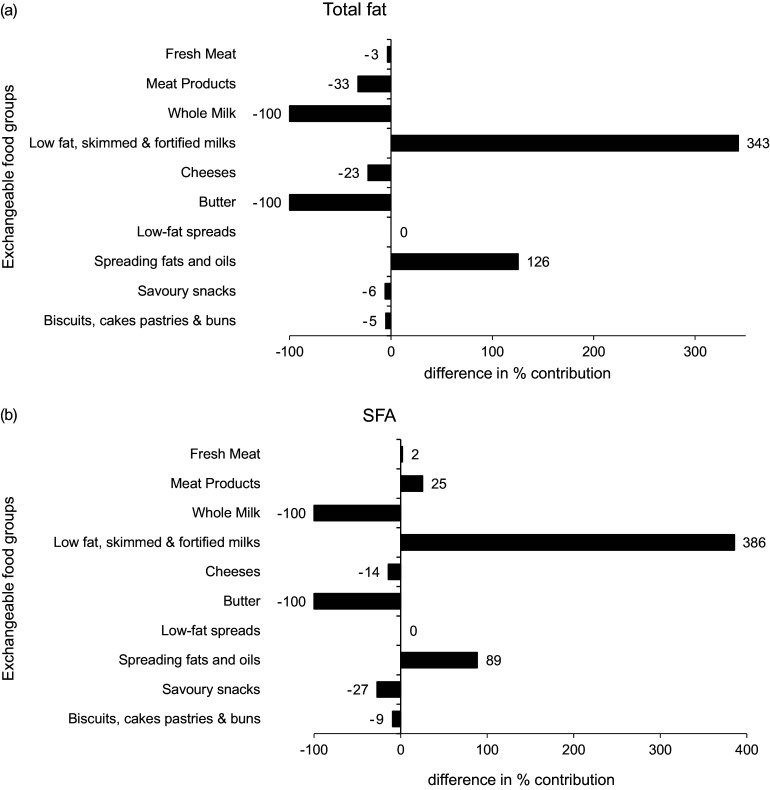
Differences in the % contribution of food categories to (a) total fat and (b) SFA intakes between baseline and food-exchange model

The differences in the percentage contribution of exchangeable food categories to total fat and SFA intakes between baseline and modelled data are illustrated in Fig. 1. Both displayed significant differences in the percentage contribution of all dairy products. Total fat and SFA contributions from ‘whole milk’ and ‘butter’ reduced by –100 %, whereas differences in the contribution of ‘low-fat, skimmed and fortified milks’ ranged from 343 to 386 %. Significant increases were evident in the contribution of ‘spreading fats and oils’, with the largest percentage difference observed in total fat (126 %), whereas SFA showed a difference of 89 %.

### The percentage of Irish children adhering to dietary fat recommendations

**Fig. 2:**
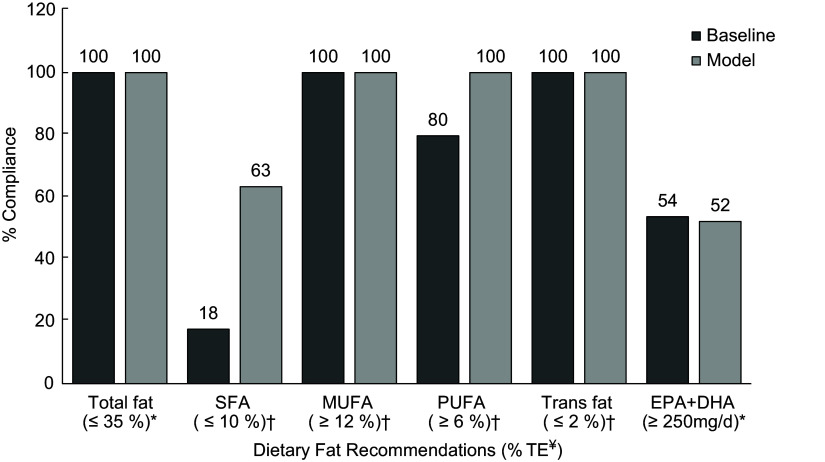
Percentage of Irish children adhering to current European Food Safety Authority and UK dietary recommendations for total fat and fatty acids between baseline and modelled intake data. *Targets from European Food Safety Authority (Dietary Reference Values)^(13)^. †Targets from the Department of Health and/or Scientific Advisory Committee on Nutrition^(9,16)^. ¥Dietary fat recommendations are presented as % TE, with the exception of EPA + DHA, which is presented as mg/d.

No changes were observed in the level of compliance to the recommendations for total fat (100 %), MUFA (100 %) and *trans*-fat (100 %) between baseline and modelled intakes (Fig. 2). Compliance with SFA recommendations significantly improved, with 63 % overall complying with the ≤ 10 % recommendation in the modelled intakes. In addition, adherence to PUFA recommendations improved, resulting in 100 % compliance within the total population. In contrast, the compliance rate of EPA and DHA slightly reduced from 54 to 52 %.

## Discussion

The present study reports the first use of nationally representative data to model the effects of a food-exchange strategy that aimed to improve the dietary fat profile in Irish children. Some of the largest and most significant contributions of SFA in high consumers were found among dairy products, namely, whole milk, cheese and butter. The inclusion of these foods in addition to meat products and snack foods within the food-exchange model proved successful, creating favourable changes to dietary fat intakes when they were replaced with lower fat alternatives. The replacement of the variety of foods containing exchangeable fat reduced children’s mean daily intakes of SFA from 14 to 11 % and improved adherence to SFA recommendations by 262 %. In addition, mean PUFA intakes increased from 6 to 7 %, and adherence to PUFA recommendations successfully improved to 100 % compliance. The findings demonstrate how modest food substitutions can effectively improve diet quality and can be achieved without compromising additional nutrient intakes or the composition of the overall diet.

The food-exchange model employed in this study proposed improvements to SFA intakes based on replacing key dietary sources of SFA with lower SFA alternatives. In essence, the foods chosen contained exchangeable fats to ensure minimal dietary disturbance and were in line with current Irish dietary guidelines and, therefore, realistic in terms of implementation. Milk and milk products accounted for a large proportion of the differences in SFA intakes between low and high consumers in the current study. These findings agree with other studies that have reported milk and milk products as major sources of SFA intakes in children and adolescents across the globe^(47–50)^. Although whole milk is one of the top contributors of SFA intakes in Irish children, milk is considered to be a nutrient-rich food, providing additional micronutrients such as Ca, iodine, vitamins A, B_6_, B_12_, riboflavin, Mg, K, P and Zn^(51)^. Therefore, it is paramount that milk remains a staple food within children’s diets as it contributes significantly to the adequate intakes of these micronutrients. The replacement of all ‘whole milk’ with a low-fat alternative ensures the continuation of habitual milk consumption and micronutrient intake while significantly reducing SFA intakes. Similar exchanges were applied for cheese, and reductions in the contribution of cheese to SFA intakes were observed, although not as large. However, there is mounting evidence to suggest that dairy SFA intake is not associated with increases in CVD risk and may potentially contain cardio-protective properties^(52–54)^. Furthermore, the concept of the dairy matrix suggests that beneficial health effects observed when consuming some dairy products may be due to the metabolic effects of whole dairy differing from its individual dairy constituents^(55,56)^. In relation to chronic disease risk, nutrition research is looking beyond the examination of isolated nutrients and investigating a whole food approach. Therefore, it is important that the whole food structure and the relationship between its components are considered^(55)^. Nevertheless, current Irish and UK dietary guidelines recommend that SFA intakes provide no more than 10 % TE and that low-fat dairy products should be chosen over full-fat^(9,45,46)^. Moreover, dairy products collectively contribute 27 % to SFA intakes in Irish children^(17)^. Thus, strategies such as the food exchange applied in this study may have more impact in improving total SFA intakes through the simple replacement of full-fat dairy products with low-fat alternatives, as it does not heavily rely on dietary behaviour change and in addition, it limits negative nutritional effects. The caveat here is that the current guidelines fail to recognise the considerable evidence that not all SFA are created equal^(57)^. Astrup and colleagues argue that the health effects of SFA vary significantly depending on the specific SFA and the specific food source and opine that by maintaining general advice to reduce total SFA, it could work against the guidelines intentions and weaken their effect on chronic disease risk^(57)^.

Although no differences were noted between low and high SFA consumers for fresh meat and meat products, our previous work has established them as significant contributors to SFA intakes among Irish children^(17)^. Therefore, in line with recommendations to replace fresh meat and meat products with leaner cuts, this food category was included in the food-exchange model. However, others have cautioned about the exchangeable fat within these products and incorporating it into such a model. The LIPGENE intervention study, which developed a food-exchange model to manipulate overall dietary fat intake in a large European adult cohort, described this as fat that was not intrinsic within a food; that is, it cannot be easily removed and replaced^(34)^. Hence, they excluded meat and meat products from their model indicating it would be difficult to manipulate the fat within these foods, despite these products significantly contributing to SFA intakes. However, the model utilised in the present study selected meat and meat product replacements that minimised additional changes to the composition of the food. For example, fatty meats were replaced with leaner cuts of the same product, and cooking methods such as frying were substituted for grilled or dry-fried alternatives. And while a significant reduction was observed in their contribution to PUFA intakes, no negative outcomes were observed for mean PUFA intakes or overall PUFA compliance. In addition, small but significant increases were observed in protein and total sugar intakes, increasing from 15·9 % TE to 16·8 % TE and 18·8 % TE to 19·3 % TE, respectively. While the increase observed in protein is most likely due to the leaner cuts providing more protein per gram, the increase observed in total sugar may be more complex. Previous research has reported a reciprocal relationship between fat and sugar intakes, including a systematic review that reported that low-sugar consumers are more likely to consume over 30 % of energy from total fat than those consuming higher levels of sugar^(58–60)^. This association is known as the ‘sugar-fat seesaw’ and indicates that elevated sugar intakes are a potential outcome when fat intake is reduced within the diet^(60)^. However, as this study made dietary modifications based on food sources of exchangeable fat, it is reasonable to suggest that the potential consequences of the sugar-fat seesaw effect have been minimised in the model diet. No differences were noted in micronutrient intakes with the exception of vitamin E. Vitamin E increased in the model diet, which may have a positive influence as this fat-soluble antioxidant can help prevent the oxidation of PUFA^(61)^. It is also an extremely effective nutrient in modulating immune function^(62)^.

To further reduce SFA intakes and improve compliance, additional food exchanges were made from a variety of snack foods such as biscuits (all kinds), popcorn, rice cakes and cream crackers. Even though some of the fat within these foods is arguably non-exchangeable, particularly for biscuits, dietary guidelines classify these products as foods high in fat, sugar and salt and are, therefore, to be limited as much as possible^(45)^. The approach taken in this study has merely replaced these products with low-SFA alternatives. Preferably, some of these foods should be excluded or intakes reduced in the children’s diets which would drastically improve their overall diet quality. However, this would require a significant change in children’s dietary behaviours. As dietary behaviours are influenced by a number of complex factors, a multitude of strategies beyond educational measures such as dietary guidelines would be required to shift consumption habits surrounding these confectionery foods at a population level^(63)^.

Previous legislative measures such as bans and mandatory reformulation at the manufacturing level have proven successful in reducing *trans*-fat intakes across Europe^(21,22)^. In Ireland, additional measures to improve Irish diets include the newly developed roadmap for food product reformulation^(64)^. Created by a dedicated subgroup of the 2016 government-approved ‘Obesity Policy and Action Plan’ known as the Reformulation Task Force, the roadmap aims to drive the food industry towards reformulating food for healthier diets^(64)^. This is one of multiple actions on obesity prevention, with the overarching aim of the Obesity Policy and Action Plan being to reverse trends in overweight and obesity and prevent the health complications of excess weight, thus reducing the overall burden for the Irish population, the health system, wider society and the economy^(64)^. In terms of SFA, one of the roadmap’s proposed targets is a 10 % reduction in the SFA content of processed foods that contribute most to SFA intakes in Ireland^(64)^. Furthermore, taxation measures have also been implemented to reduce sugar intakes as research has suggested that an increase in price for high-sugar foods can significantly influence the amount of sugar consumed^(65–67)^. Given the adverse health effects associated with elevated SFA intakes, the WHO have urged for further taxation measures to be applied to high-fat and SFA foods^(68)^. Modelling the health impacts of a fat tax in Europe, Schönbach *et al.* found that the implementation of an SFA tax could significantly reduce the prevalence of ischaemic heart disease^(69)^. In addition, they hypothesise that this would encourage the industry to replace foods high in SFA with PUFA, another recommended strategy to improve the dietary fat profile^(9,69)^. Therefore, relying on a food-exchange approach to radically reduce the consumption of high-fat confectionery foods may not be sufficient in isolation and would require additional support. Of note, SFA levels in Irish children remain above the 10 % TE threshold, irrespective of the number of food exchanges applied within the current model. This emphasises the complexity and difficulty in reducing SFA intakes to recommended levels.

As previously mentioned, one SFA replacement strategy incorporated into the food-exchange model was the replacement of butter and butter spreads containing high amounts of SFA with high PUFA spreads. This single replacement strategy, in addition to improving SFA intakes, also increased PUFA intakes with modelled intakes meeting the recommendation of over 6 % TE. However, EPA and DHA intakes remained fixed and well below the recommended 250 mg/d^(13)^. Given their known health benefits in children such as contributing to adequate brain function and cognitive development, poor intakes of EPA and DHA in Irish children need to be addressed^(70)^. The Scientific Advisory Committee on Nutrition recommends two portions of fish a week, averaging 140 g each (one white, one oily) which equates to 40 g of fish per d^(71)^. Despite ‘fish, fish products and fish dishes’ being a key source of EPA and DHA in Irish children, the average consumption is currently only 13 g/d^(72)^. While strategies to promote an increase in fish consumption is the most obvious approach to improve EPA and DHA intakes, this could prove difficult in a Western society where fish intakes are generally low^(73)^. While supplementation can play a role in increasing EPA and DHA intakes, they are not routinely recommended in healthy individuals and children, whereas food sources, particularly fish, are encouraged^(74)^. Other measures such as *n*-3 fortification have been tested, but challenges remain due to formulation issues in addition to a lack of consumer interest^(75,76)^. However, reformulation efforts have successfully enriched foods with *n*-3 such as poultry and eggs through the supplementation of animal feed^(23,24)^, which highlights other potential opportunities to improve EPA and DHA intakes through commonly consumed foods.

A key strength of the current study is applying a food-exchange modelling approach on a nationally representative dataset with the comprehensive dietary intake and composition data aiding the process of exchanging foods within the population. In addition, the food-exchange model created significant changes to children’s dietary fat profile with minimal disturbance to their dietary habits, thus increasing the model’s likelihood of success. However, due to the study’s cross-sectional design, this study is limited in evaluating the effectiveness of the food-exchange model, and additional intervention studies would be required to further evaluate the direct impact and credibility of the model.

In conclusion, the food-exchange model employed in this study has shown that targeted dietary fat strategies can effectively decrease SFA intakes as well as increase PUFA intakes in Irish children, improving their overall dietary fat profile. Moreover, if the total population conformed to the food exchanges examined in this study, it would result in dramatic increases in compliance with SFA and PUFA dietary guidelines. However, while notable improvements were made, overall SFA compliance remained inadequate, and adherence to EPA and DHA recommendations remained poor, despite improvements to total PUFA intakes. Nevertheless, this study indicates modest food-exchange methods, which make small but significant modifications to children’s diets and can improve their dietary fat profile considerably with minimal changes to overall dietary habits. It is noteworthy that one strategy cannot independently elicit behaviour change at a population level and a number of approaches delivered together would yield more effective results.

## Supporting information

O’Connor et al. supplementary material O’Connor et al. supplementary material

## Supplementary material

For supplementary material accompanying this paper visit https://doi.org/10.1017/S1368980024000971.
