# Supplementary material for: A food-exchange model for achieving the recommended dietary intakes for saturated fat in Irish children: analysis from the cross-sectional National Children’s Food Survey II
Source: Public Health Nutr. 2024 May 3;27(1):e140. doi: 10.1017/S1368980024000971 (PMC11625449; doi:10.1017/S1368980024000971)
Supplement: O’Connor et al. supplementary material [file S1368980024000971sup001.docx]

**Supplementary Data**

| **Supplementary Table 1.** Mean daily intakes of macronutrients and micronutrients across tertiles of SFA and PUFA (% TE) in Irish children | | | | | | | | | | | | | | |
| --- | --- | --- | --- | --- | --- | --- | --- | --- | --- | --- | --- | --- | --- | --- |
|  | SFA | | | | | | | PUFA | | | | | | |
|  | Lowest | | Medium | | Highest | |  | Lowest | | Medium | | Highest | |  |
|  | *n* 200 | | *n* 200 | | *n* 200 | |  | *n* 200 | | *n* 200 | | *n* 200 | |  |
|  | Mean | SD | Mean | SD | Mean | SD | *P** | Mean | SD | Mean | SD | Mean | SD | *P** |
| Energy (kcal) | 1448^a^ | 340 | 1491^ab^ | 362 | 1554^b^ | 357 | 0.374 | 1455^a^ | 318 | 1487^ab^ | 353 | 1551^b^ | 387 | 0.782 |
| **Macronutrients** |  |  |  |  |  |  |  |  |  |  |  |  |  |  |
| Protein (g) | 57.8 | 19.0 | 59.3 | 17.0 | 61.1 | 15.9 | 1.000 | 57.2 | 14.8 | 60.0 | 17.3 | 61.0 | 19.5 | 1.000 |
| Protein (%TE) | 16.0 | 3.13 | 16.0 | 2.72 | 15.8 | 2.56 | 1.000 | 15.8 | 2.50 | 16.2 | 3.06 | 15.7 | 2.82 | 1.000 |
| CHO (g) | 207^a^ | 48.8 | 198^ab^ | 49.5 | 190^b^ | 46.4 | 0.068 | 204 | 45.1 | 197 | 51.9 | 196 | 48.5 | 1.000 |
| CHO (%TE) | 54.0^a^ | 4.67 | 50.0^b^ | 4.30 | 46.1^c^ | 4.02 | <0.001 | 52.8^a^ | 4.85 | 49.7^b^ | 5.01 | 47.5^c^ | 5.04 | <0.001 |
| Total sugars (g) | 75.0 | 25.5 | 74.9 | 25.8 | 75.1 | 25.4 | 1.000 | 82.1^a^ | 25.6 | 72.4^b^ | 25.0 | 70.4^b^ | 24.6 | <0.001 |
| Total sugars (%TE) | 19.4^a^ | 4.85 | 19.0^ab^ | 5.15 | 18.1^b^ | 4.56 | 1.000 | 21.2^a^ | 4.92 | 18.2^b^ | 4.23 | 17.1^b^ | 4.55 | <0.001 |
| Total fat (g) | 47.3^a^ | 13.9 | 56.0^b^ | 15.7 | 65.5^c^ | 17.4 | <0.001 | 50.1^a^ | 14.7 | 55.6^b^ | 15.1 | 63.1^c^ | 19.4 | <0.001 |
| Total fat (%TE) | 29.3^a^ | 3.71 | 33.6^b^ | 3.45 | 37.8^c^ | 3.70 | <0.001 | 30.7^a^ | 4.56 | 33.6^b^ | 4.50 | 36.4^c^ | 4.39 | <0.001 |
| SFA (g) | 17.9^a^ | 4.89 | 23.2^b^ | 5.80 | 29.4^c^ | 7.14 | <0.001 | 22.9 | 7.79 | 23.3 | 6.97 | 24.3 | 8.04 | 1.000 |
| SFA (% TE) | 11.1^a^ | 1.37 | 14.0^b^ | 0.66 | 17.0^c^ | 1.68 | <0.001 | 14.1 | 3.04 | 14.1 | 2.75 | 14.0 | 2.50 | 1.000 |
| MUFA (g) | 20.1^a^ | 6.68 | 23.1^b^ | 7.33 | 26.3^c^ | 7.91 | <0.001 | 20.1^a^ | 6.04 | 23.1^b^ | 6.69 | 26.4^c^ | 8.88 | <0.001 |
| MUFA (%TE) | 12.4^a^ | 2.18 | 13.9^b^ | 2.20 | 15.2^c^ | 2.26 | <0.001 | 12.3^a^ | 1.98 | 13.9^b^ | 2.12 | 15.2^c^ | 2.43 | <0.001 |
| PUFA (g) | 9.12 | 3.38 | 9.41 | 3.42 | 9.56 | 3.56 | 1.000 | 6.86^a^ | 1.81 | 9.07^b^ | 2.22 | 12.3^c^ | 3.74 | <0.001 |
| PUFA (%TE) | 5.64 | 1.36 | 5.65 | 1.36 | 5.48 | 1.20 | 1.000 | 4.26^a^ | 0.53 | 5.47^b^ | 0.28 | 7.04^c^ | 0.99 | <0.001 |
| Dietary fibre (g) | 14.8^a^ | 4.87 | 14.1^ab^ | 4.27 | 13.4^b^ | 4.21 | 0.238 | 14.0 | 4.67 | 13.7 | 4.15 | 14.5 | 4.61 | 1.000 |
| **Micronutrients** |  |  |  |  |  |  |  |  |  |  |  |  |  |  |
| Vitamin A (µg) | 568^a^ | 424 | 615^a^ | 358 | 768^b^ | 507 | <0.001 | 672 | 420 | 612 | 420 | 668 | 482 | 1.000 |
| Vitamin C (mg) | 4.30 | 4.06 | 4.17 | 4.84 | 4.70 | 6.8 | 1.000 | 4.50 | 4.88 | 4.46 | 6.52 | 4.21 | 4.44 | 1.000 |
| Vitamin D (µg) | 74.7 | 61.7 | 75.4 | 101 | 74.2 | 119 | 1.000 | 73.8 | 54.2 | 71.4 | 85.3 | 78.9 | 134 | 1.000 |
| Vitamin E (µg) | 6.85 | 3.62 | 6.81 | 3.45 | 6.99 | 4.3 | 1.000 | 5.99^a^ | 3.83 | 6.70^a^ | 3.42 | 7.93^b^ | 3.89 | <0.001 |
| Vitamin B6 (mg) | 1.58 | 0.68 | 1.45 | 0.54 | 1.52 | 1.0 | 1.000 | 1.50 | 0.55 | 1.53 | 0.63 | 1.52 | 1.07 | 1.000 |
| Vitamin B12 (µg) | 4.00^a^ | 2.00 | 4.59^b^ | 1.92 | 5.35^c^ | 2.8 | <0.001 | 4.79 | 2.48 | 4.56 | 2.01 | 4.55 | 2.47 | 1.000 |
| Thiamin (mg) | 1.49 | 0.73 | 1.35 | 0.45 | 1.37 | 0.6 | 1.000 | 1.45 | 0.50 | 1.39 | 0.49 | 1.38 | 0.75 | 1.000 |
| Riboflavin (mg) | 1.44^a^ | 0.60 | 1.61^a^ | 0.63 | 1.72^b^ | 0.7 | <0.001 | 1.73^a^ | 0.70 | 1.56^b^ | 0.60 | 1.48^b^ | 0.67 | <0.001 |
| Total folate (µg) | 217 | 82.5 | 205 | 75.1 | 202 | 81.7 | 1.000 | 220^a^ | 81.0 | 205^ab^ | 71.7 | 200^b^ | 85.6 | 1.000 |
| DFE (µg) | 266 | 124 | 246 | 112 | 240 | 124 | 1.000 | 270^a^ | 122 | 247^ab^ | 108 | 235^b^ | 127 | 0.476 |
| Total Niacin (mg) | 30.1 | 10.7 | 28.5 | 9.0 | 28.1 | 7.8 | 1.000 | 27.9 | 8.06 | 29.4 | 9.82 | 29.3 | 9.83 | 1.000 |
| Sodium (mg) | 1640 | 480 | 1650 | 533 | 1745 | 502 | 1.000 | 1568^a^ | 449 | 1683^ab^ | 485 | 1784^b^ | 561 | <0.001 |
| Potassium (mg) | 1979 | 591 | 2020 | 613 | 2016 | 545 | 1.000 | 2028 | 555 | 1976 | 612 | 2011 | 582 | 1.000 |
| Calcium (mg) | 672^a^ | 235 | 804^b^ | 284 | 892^c^ | 305 | <0.001 | 857^a^ | 323 | 775^b^ | 258 | 735^b^ | 272 | <0.001 |
| Magnesium (mg) | 188 | 53.7 | 193 | 57.8 | 196 | 57.6 | 1.000 | 192 | 54.0 | 189 | 54.8 | 196 | 60.3 | 1.000 |
| Phosphorous (mg) | 926^a^ | 280 | 1005^b^ | 294 | 1078^c^ | 311 | <0.001 | 1014 | 302 | 993 | 289 | 1001 | 313 | 1.000 |
| Iron (mg) | 9.26 | 3.34 | 8.86 | 2.97 | 8.86 | 2.84 | 1.000 | 9.15 | 3.05 | 9.02 | 2.82 | 8.82 | 3.30 | 1.000 |
| Zinc (mg) | 6.71^a^ | 2.67 | 7.27^ab^ | 2.43 | 7.72^b^ | 2.38 | <0.001 | 7.14 | 2.20 | 7.39 | 2.76 | 7.23 | 2.60 | 1.000 |
| *n-* number; SD- standard deviation; % TE- percentage of total energy; CHO, carbohydrates; SFA, saturated fatty acid; MUFA, monounsaturated fatty acid; PUFA, polyunsaturated fatty acid; DFE, dietary folate equivalents ^abc^Different superscript letters indicate significant differences in mean values across tertiles (*P* <0.05). *One- way ANOVA was used with Scheffe *post* hoc test to assess difference between tertiles. Adjusted *P* value using Bonferroni correction method for multiple comparisons | | | | | | | | | | | | | | |
